# Effects of Ultrasound‐Guided Tenotomy and Debridement on Pain, Function, and Psychological Factors for Achilles Tendinopathy: A Prospective Cohort Study

**DOI:** 10.1002/jor.70071

**Published:** 2025-09-28

**Authors:** Mederic M. Hall, Ruth L. Chimenti, Jessica F. Danielson, Timothy R. Fleagle

**Affiliations:** ^1^ Department of Orthopedics & Rehabilitation and Department of Sports Medicine University of Iowa Iowa City Iowa USA; ^2^ Department of Physical Therapy & Rehabilitation Sciences University of Iowa Iowa City Iowa USA; ^3^ Institute of Clinical and Translational Science University of Iowa Hospitals and Clinics Iowa City Iowa USA

**Keywords:** Achilles tendon, minimally invasive surgical procedure, pain, tenotomy, ultrasonography

## Abstract

Ultrasound‐guided tenotomy and debridement is a minimally invasive treatment with a low risk of complications for individuals with chronic Achilles tendinopathy. Yet the benefits of this procedure on pain, function, and pain‐related psychological factors, as well as predictors of treatment success, remain understudied. A total of 56 individuals with chronic Achilles tendinopathy (mean (SD): age = 55.9 (11) years, BMI = 34.8 (8.2) kg/m^2^, women = 68%) underwent baseline ultrasonography, followed by ultrasound‐guided tenotomy and debridement, and rehabilitation. Participants reported pain (0–10), function (Foot and Ankle Ability Measure—ADL), kinesiophobia (Tampa Scale of Kinesiophobia‐17), and pain catastrophizing (Pain Catastrophizing Scale) at baseline and for a year following the procedure. Baseline pain was 6.1 (2.2), kinesiophobia was 40.8 (7.1), pain catastrophizing was 13.7 (10.2), and function was 55.9 (17.3). By 6 weeks, there were decreases in pain (mean change (95% CI): −1.9 (−1.1 to −2.6), function: 14.4 (9.3–19.5), kinesiophobia: −5 (−3.2 to −6.9), and pain catastrophizing: −7 (−4.9 to −9.1)). Patient‐reported outcomes were similar at 52 weeks (pain: −2.99 (−2.2 to −3.8), function: 25.1 (19.6–30.7), kinesiophobia: −7.5 (−6.1 to −11.4), catastrophizing: −8.5 (−6.1 to −10.8)) following the procedure. Haglund deformity (*β*: −13.1 (−0.6 to −25.5)) and intratendinous calcifications (*β*: −14.7 (−1.4 to −28.1)) were associated with smaller improvements in function. No procedure‐related complications were reported. Clinical significance: Ultrasound‐guided tenotomy and debridement for chronic Achilles tendinopathy may provide positive outcomes for pain, function, and pain‐related psychological factors at 6‐week and 1‐year follow‐up. Haglund deformity and tendon calcifications were associated with smaller improvements in function.

## 1 Introduction

Achilles tendinopathy is a common cause of posterior ankle pain and is typically treated with exercise [1, 2, 3]. Despite strong evidence supporting the use of exercise to treat Achilles tendinopathy, clinical outcomes vary widely, with up to 60% of individuals experiencing persistent pain and reduced function following these frontline treatments [4]. Open surgical procedures are possible second‐line treatments reserved for patients with symptoms lasting at least 6–12 months and unresponsive to frontline treatments [3]. These procedures while effective are associated with an increased risk of complications including infection, persistent pain, and delayed recovery [5]. In addition, certain patient populations may not be appropriate for surgical management either due to increased risk related to comorbidities or the need to return to high‐level activities in a short timeframe, such as elite athletes. This has led to the development of minimally invasive surgeries for Achilles tendinopathy [6, 7], yet there is still a need for evidence to justify its increasing use in clinical practice.

There is a need for prospective studies to investigate the outcomes of ultrasound‐guided tenotomy and debridement (USGTD) in managing pain and decreased function in individuals with Achilles tendinopathy. While prospective studies support the effectiveness of USGTD for managing frontline treatment‐resistant tendinopathies in the upper extremity [8, 9], its application in the lower extremity, including Achilles tendinopathy, remains understudied. Case studies and retrospective analysis have shown improvements in pain and function but have primarily reported patient satisfaction as their primary outcome [7, 10]. Moreover, there is a growing need to understand the impact of USGTD on pain‐related psychological factors [11]. Fear of movement and pain catastrophizing are associated with worse Achilles tendinopathy symptoms [12] and worse outcomes following multiple orthopedic surgical procedures [13, 14, 15]. Rehabilitation, including tendon‐loading exercise and education, reduces pain‐related psychological factors [16]. However, little is known of the effects of surgical procedures on these pain‐related psychological variables for individuals with Achilles tendinopathy. Prospective research on USGTD for Achilles tendinopathy is needed to not only fill a gap in research on the effects on pain and function but also on pain‐related psychological factors.

Identifying patients who will benefit most from second‐line treatments, such as USGTD, is crucial for individualized care and treatment efficiency. Ultrasound imaging is currently a recommended imaging modality in tendinopathy [17] and is commonly used in clinical practice to inform procedures [3] and predict treatment effects across multiple musculoskeletal conditions [18, 19, 20]. Previous investigations that aimed to identify imaging factors predictive of treatment success have identified that a larger degree of tendon thickening and nonuniform tears identified using MRI in the insertion were associated with not responding to frontline rehabilitation [21, 22]. Additionally, the presence of hyper‐ and hypoechoic areas in the tendon has also been associated with worse treatment outcomes [23], while changes in neovascularization are associated with improved treatment outcomes following rehabilitation [24]. Identifying ultrasound imaging predictors of treatment outcomes will assist clinicians in selecting appropriate patients for USGTD procedures and more personalized care for tendinopathy.

The primary objective of this study was to investigate the short‐term outcomes of USGTD followed by postprocedure rehabilitation in treating pain, function, and pain‐related psychological factors in individuals with chronic Achilles tendinopathy. It was hypothesized that USGTD would lead to improvements in pain, self‐reported function, and reductions in pain‐related psychological factors, including fear of movement and pain catastrophizing, at short‐term follow‐up (6 weeks). In addition, an exploratory objective was to assess the long‐term outcomes of USGTD at 52 weeks and to assess self‐reported self‐efficacy and satisfaction following USGTD. Additionally, the identification of ultrasound imaging predictors for treatment outcomes following USGTD was explored.

## 2 Methods

### 2.1 Participants

Individuals with chronic Achilles tendinopathy who were electing to have USGTD were recruited from a single sports medicine clinic to participate in this prospective study (Level 4 evidence) from June of 2019 through September of 2021. Participants were eligible to participate if they were 18 years or older, had chronic Achilles tendinopathy (> 3 months), and had failed to respond to usual conservative care as determined by the evaluating physician. This typically included a trial of tendon loading exercise, activity modification, heel lifts/footwear modification, and oral medications for at least 12 weeks. Participants were excluded if they were over the age of 90 or not fluent in English. All study procedures were approved by the local Institutional Review Board (IRB) at the University of Iowa, United States of America, prior to the data collection. All participants provided informed consent prior to enrolling in the study. Due to limited data on the effects of USGTD in Achilles tendinopathy at the time of study design, a priori sample size calculation was not performed. Sample size was determined by feasibility and clinical caseload.

### 2.2 Baseline Imaging

**Figure 1:**
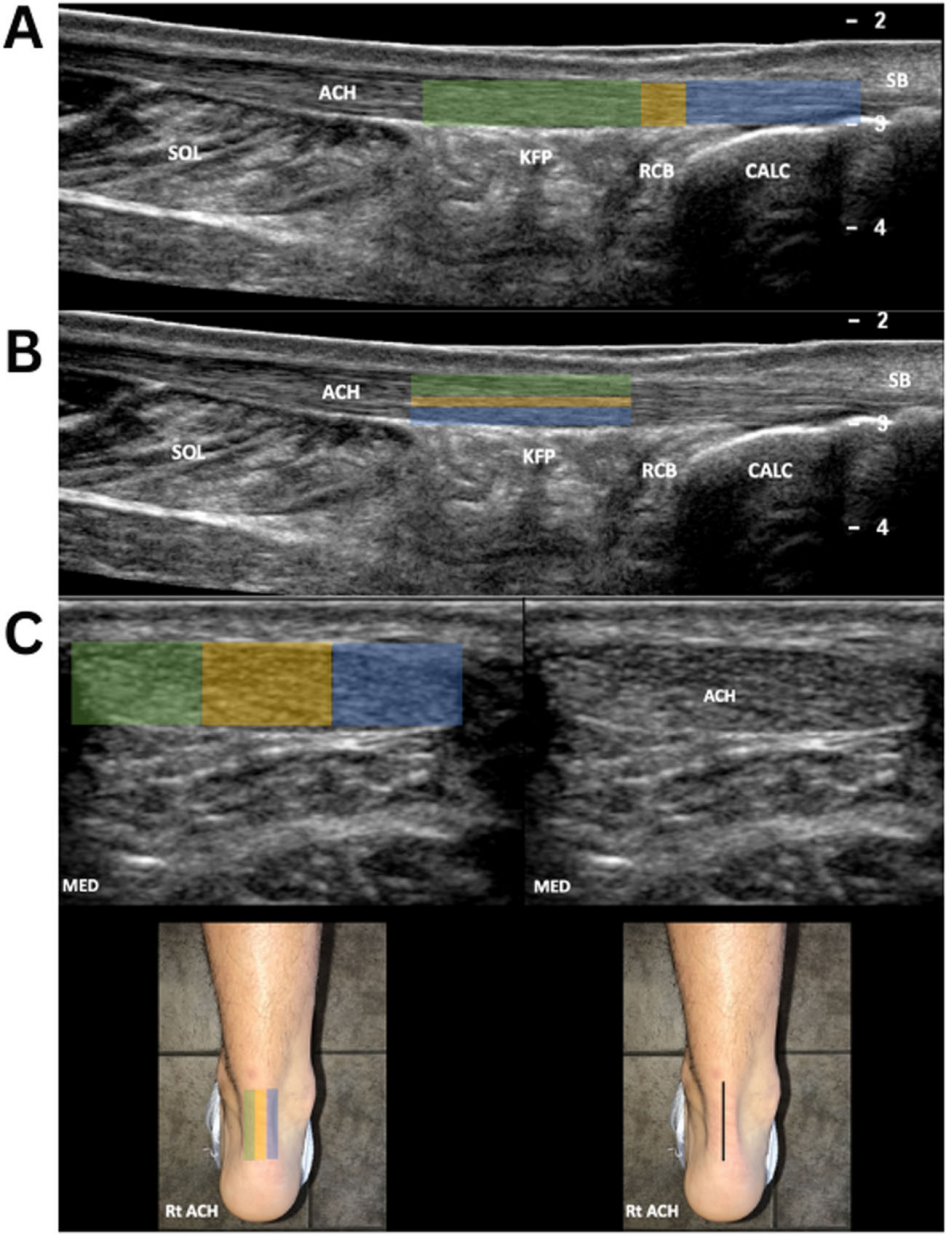
Depiction of the imaging sequence. (A) Imaging location for midportion (green), retrocalcaneal bursa level (yellow), and insertion (blue). (B) Sections of the tendon from superficial (green) to middle (yellow), and deep (blue). (C) Location of medial (green), central (yellow), and lateral (blue) portions of the tendon. A line is drawn bisecting the Achilles tendon in the long axis. For medial images, the transducer is placed medial to the bisecting line. The transducer is placed lateral to the bisecting line for lateral images. ACH, Achilles tendon; CALC, calcaneus; KFP, Kager fat pad; MED, medial; RCB, retrocalcaneal bursa; Rt, right; Sol, soleus muscle.

Each participant underwent a standardized ultrasound examination by a single fellowship‐trained sports medicine physician with extensive experience in diagnostic ultrasonography (M. M. H.). Images were acquired while the participant was prone and the ankle was placed in a resting neutral position with the foot hanging free off the examination table. A high‐frequency linear array transducer (Canon Aplio i800) was used to acquire B‐mode and Doppler still images and cine loops following a standardized protocol (Figure 1). Images were uploaded to PACS (Carestream) using the usual clinical workflow, where they were available for viewing through the electronic medical record.

### 2.3 Intervention

USGTD was performed by the same experienced sports medicine physician (M. M. H.) with postgraduate training in ultrasound‐guided procedures, including USGTD. The procedure has been described previously [10]. In short, local anesthesia was applied to the Achilles tendon and surrounding tissues. If bone work was to be performed, nerve blocks of the sural nerve and medial calcaneal sensory nerve were utilized in addition to local anesthesia. A small incision was created over the posterior aspect of the Achilles tendon. An ultrasonic cutting device (Tenex Health TX, Trice Medical, Malvern, PA) was introduced into the incision. The device was used to debride the posterior bursa and paratenon before being introduced into the tendon proper. Using ultrasound guidance, pathologic areas of the tendon, including any calcifications, were debrided. When indicated, resection of pathologic bone was performed, including removal of posterior calcaneal enthesophytes and a limited resection of the posterior superior calcaneus consistent with Haglund's deformity. The retrocalcaneal bursa was also debrided as indicated. Following the procedure, the incision was closed using a steri‐strip.

### 2.4 Postprocedure Rehabilitation

Immediately following the USGTD procedure, patients were given a referral for physical therapy and started a rehabilitation program. For the first 2 weeks, patients were instructed to weight bear as tolerated in a walking boot and to use assistive devices until walking in the boot was pain‐free. Once walking and range of motion were pain‐free, participants progressed with strengthening exercises and functional activities as pain allowed. Most patients were released to full activity by 3–4 months based on functional status and progression through the rehab protocol [25].

#### 2.4.1 Outcome Measures

##### 2.4.1.1 Pain

Pain was assessed using an 11‐point numeric pain rating scale (NRS) where participants were asked to rate their worst pain in the last week between 0 and 10 where 0 is *no pain* and 10 is the *worst imaginable pain*. The measure of the worst pain in the last week was selected as tendinopathy is typically exacerbated by activity and thus average pain may underestimate an individual's pain experience and is suitable as a patient‐reported outcome in clinical trials [26]. Pain was collected at 2, 6, 12, 26, and 52 weeks following the procedure. The minimal clinically important difference (MCID) for pain was considered 2/10 or a 20% improvement from baseline [27].

##### 2.4.1.2 Function

To assess patient‐reported function, participants were asked to complete the Foot and Ankle Ability Measure (FAAM) Activities of Daily Living (ADL) subscore. The FAAM‐ADL is scored from 0 to 81 with higher scores representing increased function. The MCID for the ADL subscore is 8 points [28]. In addition, the Patient‐Reported Outcome Information System (PROMIS)—Physical Function (PF) computer adaptive test was also collected to assess function. The PROMIS‐PF measure has previously been used to assess function in Achilles tendinopathy [16, 29], and is reliable and valid in foot and ankle conditions [30]. The PROMIS‐PF was scored and reported on a *t*‐score scale, where 50 represents the population average for physical function and the standard deviation (SD) is 10. The MCID ranges from 7.6 to 8.4 [31].

##### 2.4.1.3 Pain‐Related Psychological Factors

Fear of movement (i.e., kinesiophobia) was assessed using the Tampa Scale of Kinesiophobia (TSK‐17). The TSK is scored 17–68 with higher scores representing higher fear of movement [32]. The MCID for the TSK‐17 in individuals with AT has not been established though scores > 37 are considered clinically elevated in other musculoskeletal pain conditions [33]. Pain catastrophizing was assessed using the Pain Catastrophizing Scale (PCS) which is scored 0–52 [34]. Scores > 30 are considered elevated and the MCID has been reported to be between 7 and 8 for musculoskeletal pain conditions [34, 35]. PROMIS—Self‐Efficacy of Managing Symptoms (SE) and Satisfaction with Social Roles (SR) were used to assess self‐efficacy and satisfaction with social roles, respectively. The PROMIS‐SE and PROMIS‐SR were both reported in *t*‐scores similar to the PROMIS‐PF. Scores > 1 SD away from the mean (40 or 60) would be considered high or low, respectively.

##### 2.4.1.4 Patient Satisfaction

Patients were asked to complete both a modified Global Rating of Change (GROC) and patient acceptable symptom state (PASS) at each follow‐up. The modified GROC is scored from 0 (*None*—No good at all, ineffective treatment) to 3 (*Excellent—*Ideal response—virtually pain‐free) [36]. The PASS is answered as yes or no to the question “Taking into account all the activities you have during your daily life, your level of your tendon/fascia pain, and also your functional impairment, do you consider that your current state is satisfactory?” [37, 38] For the purpose of this study, the PASS was used to directly assess if participants were satisfied with the USGTD procedure. We interpreted an answer of yes as the participant was satisfied with the outcomes of the procedure.

##### 2.4.1.5 Imaging Measures

A single grader with over 10 years of clinical experience using ultrasound imaging (M. M. H.) assessed each tendon. Achilles tendon thickness was assessed using the digital calipers at the insertion of the tendon, the retrocalcaneal portion of the tendon, and the midportion of each tendon (Figure 1). The maximum thickness of the Achilles tendon was analyzed as a predictor. The presence of retrocalcaneal bursitis, superficial bursitis, and neovascularization was determined using low flow optimized Doppler (Superb Micro‐vascular Imaging, Canon Medical) as well as the presence of fluid and edema within the bursal tissue layers. The presence of intratendinous calcifications, posterior calcaneal spur, and Haglund's deformity was assessed using multiple views on B‐mode ultrasound images. The presence of these six signs of pathology were analyzed as dichotomous variables in the analysis. The tendon was divided into a standardized 2 × 3 × 3 three‐dimensional grid to allow for assessment of the location of pathologic tissues either in the medial, central, or lateral areas and the superficial, middle, or deep aspects of the tendon in both the midportion, or insertion of the tendon (Figure 1). The presence of pathology in each of these regions was recorded. Any region of focal hypoechogenicity, fibrillar heterogeneity, or fibrillar disruption was considered abnormal. To indicate how widespread the pathology was within the tendon, a composite score ranging from 0 to 9 was computed by summing together the number of tendon subregions with pathology.

##### 2.4.1.6 Statistical Analysis

The normality of continuous variables was examined using the quantile–quantile plot. For continuous variables, the mean and SD or median and interquartile range were computed, as appropriate based on the distribution of data. Linear mixed‐effects models were used to analyze the main effect of time on our primary outcomes pain, physical function, kinesiophobia, pain catastrophizing, as well as exploratory outcomes PROMIS‐PF, PROMIS‐SE, and PROMIS‐SR following USGTD from baseline to 52 weeks. Covariates included baseline levels of the dependent variable and tendinopathy type (i.e., insertional or midportion).

To assess potential imaging features as predictors of treatment success for pain and function, two separate multiple linear regression models were built using purposeful model selection. Univariate analysis between each imaging predictor assessed during the study and change in Pain or FAAM‐ADL was performed. On one occasion, a participant was missing baseline FAAM‐ADL in this instance their FAAM‐ADL from their 6‐week visit was used as a baseline to calculate the change in FAAM‐ADL. Imaging predictors with *p* < 0.3 were included in model selection. Models containing each imaging predictor combination were compared using the adjusted *R* ^2^ where higher *R* ^2^ values represent better model fit.

Patient satisfaction was assessed by calculating the percentage of participants who responded to each category within the modified GROC and the percentage of participants who responded to each possible answer for the PASS.

##### 2.4.1.7 Missing Data

This study prospectively followed participants through their clinical care and thus there is missing data as participants were lost to follow‐up. At 6 weeks, there was missing data from 4 (7%) participants, at 12 weeks, there was missing data from 12 (21%) participants, and at 52 weeks, 16 (29%) participants. The 26‐week outcomes were assessed in only a portion of the sample (*n* = 37; 38%) from this data was missing from 3 (15%) participants. Linear mixed effects models are robust to missing data under the assumption that the data is missing at random or missing completely at random. Little's test was used to assess this assumption (*p* > 0.05) suggesting data is missing completely at random. The analysis of imaging measures and patient subjective reported improvements were limited to subpopulations of the participants. There were no differences in clinical outcomes for pain, function, and pain‐related psychological variables between those who had baseline imaging who did not. Clinical outcomes were also similar between participants who completed the modified GROC and PASS and those who did not (Table S1).

## 3 Results

### 3.1 Participants

A total of 56 participants participated in the study. The mean time between baseline and intervention was 1 month (SD: 1.1). Demographics and baseline pain, function, kinesiophobia, pain catastrophizing, self‐efficacy, and satisfaction with social roles are listed in Table 1.

**Table 1:** Demographic and baseline data are presented as mean (standard deviation) or number of participants as appropriate.

Participants (*N* = 56)	
Age (years)	55.9 (11)
BMI (kg/m^2^)	34.8 (8.2)
Sex (*n*)	38F/18M
Tendinopathy type (*n*)	Insertional: 48 Midportion: 8
Worst pain (NRS: 0–10)	6.1 (2.2)
Function (FAAM‐ADL)	55.9 (17.3)
Kinesiophobia (TSK‐17)	40.8 (7.1)
Pain Catastrophizing (PCS)	13.7 (10.2)
PROMIS Physical Function (*t*‐score)	40.4 (5.5)
PROMIS—Self‐Efficacy of Managing Symptoms (*t*‐score)	43.2 (5.1)
PROMIS Satisfaction in Social Roles (*t*‐score)	41.6 (6)

Abbreviations: FAAM‐ADL, Foot and Ankle Ability Measure—Activity Daily living; PCS, Pain Catastrophizing Scale; PROMIS, Patient‐Reported Outcome Measure Information System; NRS, numeric rating scale; TSK‐17, Tampa Scale of Kinesiophobia.

### 3.2 Pain

Estimates of pain at each time point are presented in Table 2. At 2 weeks, pain had decreased by (mean change (95% CI; *p* value): −3.3/10 NRS (−4.1 to −2.6; *p* < 0.001)). This decrease was similar at the primary endpoint of 6 weeks −2.03 (−2.7 to −1.3; *p* < 0.001), and at 12 weeks (NRS: −2.9 (−3.6 to −2.1; *p* < 0.001)), 26 weeks: −2.8 (−3.9 to −1.8; *p* < 0.001) and 52 weeks −3.0 (−3.8 to −2.3; *p* < 0.001). There was no effect of tendinopathy type *β* = −0.01 (−1.2 to 0.8, *p* = 0.77).

**Table 2:** Mean value for each outcome over time presented as estimates from linear mixed effects models, 95% confidence intervals, and *p* values comparing change from baseline.

Visit	Estimate	95% confidence interval	*p*
Worst Pain (NRS)			
Baseline (*n* = 56)	6.1	5.5–6.7	—
2 weeks (*n* = 55)	2.8	2.2–3.4	< 0.001
6 weeks (*n* = 52)	4.1	3.5–4.7	< 0.001
12 weeks (*n* = 44)	3.2	2.6–3.9	< 0.001
26 weeks (*n* = 34)	3.3	2.3–4.2	< 0.001
52 weeks (*n* = 40)	3.1	2.4–3.7	< 0.001
FAAM‐ADL			
Baseline (*n* = 55)	56.2	51.8–60.5	—
6 weeks (*n* = 52)	70.7	66.3–75.1	< 0.001
12 weeks (*n* = 44)	79.5	74.4–84.4	< 0.001
26 weeks (*n* = 34)	80.9	73.9–87.9	< 0.001
52 weeks (*n* = 40)	81.3	76.4–86.2	< 0.001
TSK‐17			
Baseline (*n* = 56)	41.1	39.6–42.6	—
6 weeks (*n* = 52)	35.9	34.4–37.4	< 0.001
12 weeks (*n* = 44)	33.5	31.9–35.0	< 0.001
26 weeks (*n* = 34)	32.3	29.8–34.8	< 0.001
52 weeks (*n* = 40)	33.6	31.9–35.3	< 0.001
PCS			
Baseline (*n* = 56)	13.9	12.1–15.6	—
6 weeks (*n* = 52)	6.7	4.9–8.5	< 0.001
12 weeks (*n* = 44)	6.1	4.2–7.9	< 0.001
26 weeks (*n* = 34)	3.8	0.8–6.9	< 0.001
52 weeks (*n* = 40)	5.3	3.2–7.3	< 0.001
PROMIS–Physical Function (*t*‐score)			
Baseline (*n* = 56)	40.4	38.7–42.1	—
6 weeks (*n* = 52)	41.4	39.7–43.1	0.352
12 weeks (*n* = 44)	45.5	43.7–47.3	< 0.001
26 weeks (*n* = 34)	46.9	44.4–49.4	< 0.001
52 weeks (*n* = 40)	45.0	43.1–46.9	< 0.001
PROMIS–Self‐Efficacy for Managing Symptoms (*t*‐sore)			
Baseline (*n* = 56)	42.9	40.7–45.3	—
6 weeks (*n* = 52)	46.4	44.1–48.7	0.011
12 weeks (*n* = 44)	49.8	47.5–52.2	< 0.001
26 weeks (*n* = 34)	52.3	49.0–55.4	< 0.001
52 weeks (*n* = 40)	51.4	48.8–54.0	< 0.001
PROMIS–Satisfaction with Social Roles (*t*‐score)			
Baseline (*n* = 56)	41.2	38.5–43.9	—
6 weeks (*n* = 52)	45.5	42.8–48.3	0.007
12 weeks (*n* = 44)	50.8	47.9–53.6	< 0.001
26 weeks (*n* = 34)	53.2	49.3–57.1	< 0.001
52 weeks (*n* = 40)	52.6	49.5–55.6	< 0.001

Abbreviations: FAAM‐ADL, Foot and Ankle Ability Measure—Activity Daily living; NRS, numeric rating scale; PCS, Pain Catastrophizing Scale; PROMIS, Patient‐Reported Outcome Measure Information System; TSK‐17, Tampa Scale of Kinesiophobia.

### 3.3 Function

Patient‐reported function on the FAAM‐ADL increased by a score of (mean change (95% CI; *p* value) 14.5 (9.3–19.8; *p* < 0.001) at 6 weeks and was similar at 12 weeks FAAM‐ADL: 23.4 (17.8–28.9; *p* < 0.001)), 26 weeks (24.8 (17.3–32.9; *p* < 0.001)), and 52 weeks: 25.1 (19.65–30.7; *p* < 0.001). There was no significant effect of tendinopathy type on FAAM‐ADL scores following USGTD *β* = −3.4 (−12.4 to 5.6; *p* = 0.45).

PROMIS‐PF scores did not increase in the first 6 weeks (mean change (95% CI; *p* value): 0.9 (−1.1 to 2.9; *p* = 0.35)), but were higher than baseline scores at 12 weeks: 5.1 (3.0 to 7.2; *p* < 0.001), 26 weeks: 6.5 (3.8 to 9.2; *p* < 0.001) and 52 weeks: 4.6 (2.4 to 6.8; *p* < 0.001) (Table 2). There was an effect of tendinopathy type on PROMIS‐PF scores (*β* = −4.7 (−8.7 to −0.7; *p* < 0.05)) showing individuals with midportion tendinopathy had on average a smaller increase in PROMIS‐PF than those with insertional tendinopathy.

### 3.4 Kinesiophobia

Kinesiophobia decreased at 6 weeks (mean change (95% CI; *p* value): −5.2: (−7.1 to −3.4; *p* < 0.001)), and these reductions were similar at 12 weeks: −7.7 (−9.6 to −5.7; *p* < 0.001), 26 weeks: −8.9 (−11.6 to −6.12; *p* < 0.001), and 52 weeks: −7.6 (−9.6 to −5.5; *p* < 0.001). The effects of tendinopathy type were not significant *β* = 2.6 (0.25–5.4; *p* = 0.07).

### 3.5 Pain Catastrophizing

Pain catastrophizing decreased at 6 weeks (mean change (95% CI; *p* value): −7.1 (−9.3 to −4.9; *p* < 0.001)), and was similar at 12 weeks: −7.8 (−9.9 to −5.5), 26 weeks: −9.9 (−13.3 to −6.7; *p* < 0.001), and at 52 weeks −8.5 (−10.9 to −6.2; *p* < 0.001). There was no effect of tendinopathy type on decrease in pain catastrophizing *β* = 2.27 (−1.1 to 5.7; *p* = 0.19).

### 3.6 Self‐Efficacy

PROMIS‐SE scores had increased at the 6‐week time point (mean change (95% CI; *p* value): 3.5 (0.8–6.1; *p* < 0.001)). This increase was similar at 12 weeks (6.8 (4.1–9.5; *p* < 0.001)), at 26 weeks 9.3 (5.8–12.8; *p* < 0.001), and at 52 weeks following the procedure 8.44 (5.5–11.3; *p* < 0.001). There was no effect of tendinopathy type on the PROMIS‐SE scores *β* = −4.38 (−9.71 to 0.95; *p* = 0.1).

### 3.7 Satisfaction of Social Roles

PROMIS‐SR scores had increased at 6 weeks (mean change (95% CI; *p* value): 4.31 (1.2 to 7.4; *p* < 0.05)) and was similar at 12 weeks (9.6 (6.38 to 12.7; *p* < 0.001)), 26 weeks 11.97 (7.8 to 16.11; *p* < 0.001) and 52 weeks (11.36 (7.94 to 14.8 *p* < 0.001)). The effect of tendinopathy type was not significant *β *= −1.99 (−8.33 to 4.36; *p* = 0.54).

### 3.8 Symptoms Satisfaction and Perceived Improvement

The modified GROC and PASS were completed by 39 participants following the intervention. Data from the final follow‐up time for each participant were analyzed for subjective improvement. All participants reported some effect of the intervention on both the modified GROC and the PASS. On the modified GROC a majority of participants 56.4% (*n* = 22) reported there was a satisfactory effect but continued to have occasional episodes of pain or stiffness. In total, 20.5% (*n* = 8) reported they had the ideal response and were virtually pain‐free; 23.1% (*n* = 9) reported that there was a small effect but it was unsatisfactory. As for the PASS a majority of participants 53.8% (*n* = 21) reported their symptom levels were acceptable. Of the participants who completed the PASS, 46.2% (*n* = 18) reported their symptoms were unacceptable at their last follow‐up.

### 3.9 Imaging

Baseline imaging findings are presented in Table 3. Participants on average had thickened tendons (mean (SD): 0.934 (0.22) cm), and neovascularization was present in a large portion of participants (94.4%). No ultrasound imaging findings were significantly associated with changes in pain following the USGTD. The only variable selected for the pain model was superficial bursitis (*β* = 1.1; *p*: 0.231) (Table S2). A final model with both the presence of Haglund deformity (*β* = −13.05; *p*: 0.04) and calcifications in the tendon (*β* = −14.74; *p*: 0.03) were associated with smaller improvements in the FAAM‐ADL. The model containing both the presence of Haglund deformity and tendon calcifications was significant (*p*: 0.017; adjusted *R* ^2^: 0.13). The presence of Haglund deformity is associated with 13 points smaller improvement in FAAM‐ADL, and the presence of tendon calcification is associated with 14 points less improvement in function. Results from each imaging variable in the univariate analysis are presented in Table S2. Of the 56 participants enrolled in the study, 54 participants (96.4%) had full baseline imaging available for analysis (Table 3).

**Table 3:** Mean (SD) for tendon thickness and number of widespread pathology locations (1–9) at baseline imaging.

Imaging variable	Mean (SD) or *n* (%)
Tendon thickness	0.934 (0.22) cm
Widespread pathology	6.49 (2.29) composite score
Haglund deformity	28 (51.8%)
Tendon calcifications	36 (66.6%)
Neovascularization	51 (94.4%)
Superficial bursitis	37 (68.5%)
Retrocalcaneal bursitis	25 (46.3%)

*Note:* Number (%) of participants for the presence of ultrasound imaging findings.

### 3.10 Adverse Events

No procedure‐related complications were reported by participants throughout the duration of the study.

## 4 Discussion

This prospective cohort study on USGTD to treat chronic Achilles tendinopathy supported the hypothesis that USGTD was associated with positive short‐term (6 weeks) outcomes including improving pain, function, and addressing several pain‐related psychological factors. The current study found that the clinically relevant improvements in pain and function in the short term (6 weeks). Exploratory analyses indicated that outcomes were similar at long‐term follow‐up (12 weeks to 1 year) after USGTD. Additionally, there were improvements in pain‐related psychological factors, including kinesiophobia, pain catastrophizing, self‐efficacy, and social roles, which were also similar at long‐term follow‐up. Exploratory analyses also found that individuals who present with Haglund deformity, and tendon calcifications on ultrasound imaging at baseline have less improvements in function following USGTD compared with those who do not. These findings inform the discussion of expectations for patients electing to proceed with USGTD for various subtypes of AT.

The findings of this study are consistent with previous studies on USGTD for Achilles tendinopathy and upper extremity tendinopathies. A recent retrospective chart review found a decrease in pain from moderate/daily pain to mild/occasional pain in both the short‐term (6 weeks) and long‐term (12 weeks) [10]. Additionally, the only other prospective study to assess USGTD in the management of Achilles tendinopathy found a decrease of 5.8/10 NRS at 1‐year follow‐up, though Achilles tendinopathy represented 26% of the sample [39]. This is consistent with the current findings of clinically meaningful decreases in pain from an average pain of 6/10 NRS to 3/10 NRS that was maintained at 1‐year follow‐up. Other prospective studies investigating the use of USGTD in upper extremity tendinopathies have found similar improvements in pain and patient‐reported outcomes in medial and lateral elbow tendinopathies, which found a decrease in pain of 3–5 points (NRS, 0–10 scale) [8, 40]. In addition, these improvements are smaller but similar to the improvements in pain from other second‐line treatments, including open surgical procedures for insertional which range from 4/10 to 6/10 on a NRS [41, 42], and midportion tendinopathy which range from 50/100 to 70/100 on a visual analog scale [43].

Additionally, the improvements in patient‐reported physical function throughout the course of the current study are consistent with previous findings for USGTD and open surgical procedures. Previous studies of USGTD for insertional Achilles tendinopathy have also found at follow‐up that patients had low disability [44] and improvements in physical function [10]. This is consistent with the findings of the current study, which found FAAM‐ADL and PROMIS‐PF scores improve toward normative values following the procedure. In clinical studies on surgical treatments for Achilles tendinopathy, patient‐reported outcome measures assessing function are often included as secondary outcomes. Function as measured in the American Orthopedic Foot and Ankle Score (AOFAS)‐hindfoot score has been shown to increase between 45% and 61% following invasive surgical procedures [41, 42, 45]. These are comparable to the improvements on the FAAM‐ADL in the current study which on average was 45%. These findings indicate that USGTD may provide clinically meaningful reductions in Achilles tendinopathy pain and improve function for patients who do not benefit from front‐line treatments.

Both kinesiophobia and pain catastrophizing decreased throughout the course of the study, however, kinesiophobia only decreased from an elevated level (TSK > 36) of fear of movement to a moderate level (TSK 29–35), on average [46]. This is similar to the 5–8‐point decrease in TSK‐17 scores reported for Achilles tendinopathy in response to exercise and education, where the scores remain at moderate levels (TSK 29‐35) [16]. Pain catastrophizing at baseline (mean: 13.7 (SD: 10.2)) was higher than PCS scores previously reported in Achilles tendinopathy (11.7 (7.5)) [16], and slightly lower than PCS scores reported for shoulder tendinopathy (16 (12)) [47]. This could be due to differences in populations seeking frontline treatment, those seeking surgical procedures, or differences in upper extremity and lower extremity tendinopathies. Thus, the current findings are supported by the previous literature and importantly add to our understanding of how secondary interventions may affect pain‐related psychological variables. Treatments targeting the peripheral mechanisms of Achilles tendinopathy pain through procedures (i.e., injections and surgical procedures) may not be sufficient to completely reduce kinesiophobia. In contrast, pain catastrophizing may be well addressed through interventions targeting peripheral mechanisms of Achilles tendon pain through exercise, injections, or surgery. Future research is needed to assess the impact of these postprocedure rehabilitation strategies on pain‐related psychological factors.

The current study found that the presence of Haglund deformity and tendon calcifications were associated with lower improvements in function following USGTD. While tendon morphology and neovascularization have previously been demonstrated to be associated with treatment outcomes [21, 22], in the current study, only Haglund deformity and tendon calcification were associated with treatment response and not tendon thickness, tendon hypoechoic pathology, or neovascularization in the tendon. These differences with the previous literature may be due to methodological differences between the current study and previous literature. This includes different imaging modalities (MRI vs. Ultrasound) and treatments used in the analysis (rehabilitation vs. surgical). Additionally, in contrast with prior studies using radiographic assessment of calcaneal morphology, which found no association with treatment outcomes [48], our study found that the presence of a Haglund deformity was associated with less improvements in function. Despite not being associated with USGTD outcomes this study describes a novel composite score of tendon pathology which may be useful for future studies investigating Achilles tendon pathology. Our findings highlight the possible limitations of the USGTD in managing bony pathology both within the tendon and on the calcaneus. Clinicians should consider the use of ultrasound to assess calcaneal morphology and the presence of tendon calcifications prior to the use of USGTD to treat chronic Achilles tendinopathy.

Previous research on the effects of USGTD in treating Achilles tendinopathy has reported success rates between 70% and 77% based on patient report [10, 44]. The exploratory objective of the current paper in part supports these previous results. Despite improvements in pain, function, pain‐related psychological factors, and 76% of participants reporting they were satisfied with the procedure, only 56% of participants reported their symptoms state were acceptable at their final follow‐up. This may be attributed to participants who were lost to follow‐up (10/39; 25%) due to not presenting to the clinic postprocedure due to improved symptoms. In addition, the study found on average clinically meaningful improvements in pain, and function, however, there is significant variability in individual responses following the procedure and most participants continued to have small amounts of pain even at long‐term follow‐up (1 year) following the procedure. This may result in a smaller percentage of participants who reported acceptable symptom levels. This finding may provide insight into patient‐oriented treatment goals of further reducing pain, and that even small amounts of Achilles tendinopathy pain may be unacceptable to patients. Importantly, the current study had no procedural adverse events. Future research should consider patient preference for procedures while considering both patient satisfaction and procedural complications. For open surgical Achilles tendon debridement, complication rates can be up to 30% [5]. Clinicians and researchers should weigh any possible benefits of open debridement procedures with possible increased risk of complications. Patient preferences may differ depending on the clinical context and personal goals for treatments. This may lead to more personalized treatment options weighing both procedural benefits from both USGTD and traditional surgical procedures and risks with likely higher possible complication rates with traditional open procedures.

### 4.1 Strengths and Limitations

This prospective longitudinal study builds on the previous literature, which has focused on retrospective analyses and case series. Yet a key limitation of the current cohort study without a control group is the inability to compare the effectiveness of USGTD to a placebo or other second‐line treatments, including open and endoscopic surgical procedures. Future comparative effectiveness trials are needed to test the effectiveness of USGTD compared with other second‐line treatment options. Another limitation in our imaging biomarker data is lack of quantification of intratendon calcific burden. Many of the included patients had a large calcific tendon burden and it may be that there is a threshold above which USGTD becomes less effective. Future research should aim to determine if differences are noted in outcome based on the quantification of calcification within the tendon. Additionally, due to the clinical context of our data collection, it was not feasible to collect plantar flexor endurance following USGTD. Future research should examine performance‐based outcomes for USGTD.

A strength of this study is that it captured a clinically relevant, care‐seeking population and, to our knowledge, is the largest prospective study on USGTD for Achilles tendinopathy to date. However, a key limitation of the study is its reliance on a convenience sample, which likely resulted in insufficient statistical power for secondary analyses, including detecting long‐term improvements and identifying imaging‐based predictors of outcomes. Additionally, the study had a high amount of missing data at 26 weeks (61%) and 52 weeks (29%) and so long‐term outcomes should be interpreted with caution. Moreover, the findings of this study are specific to individuals seeking invasive treatment for Achilles tendinopathy and may not be generalizable to the broader population of individuals with this condition.

## 5 Conclusions

The results of the current study demonstrate that clinicians may consider the use of USGTD followed by rehabilitation as a second‐line treatment option to improve pain and physical function, and address pain‐related psychological factors in individuals with chronic Achilles tendinopathy who have failed to respond to frontline treatments. Ultrasound imaging for the presence of intratendinous calcifications and the presence of Haglund deformity should be considered to identify patients who may have smaller improvements in function following USGTD.

## Author Contributions

Mederic M. Hall and Ruth L. Chimenti made contributions to research design, the acquisition, analysis, and interpretation of data. Jessica F. Danielson assisted in the acquisition of data. Timothy R. Fleagle assisted in the analysis and interpretation of data and writing the original draft of the paper. All authors contributed to the revisions and approval of the final manuscript.

## Ethics Statement

All study procedures were approved by the local Institutional Review Board (IRB) at the University of Iowa, United States of America, prior to the data collection.

## Consent

All participants provided informed consent prior to enrolling in the study.

## Supporting information

Supplementary Tables 71125.
