# Supplementary material for: Effects of Ultrasound‐Guided Tenotomy and Debridement on Pain, Function, and Psychological Factors for Achilles Tendinopathy: A Prospective Cohort Study
Source: J Orthop Res. 2025 Sep 28;44(2):e70071. doi: 10.1002/jor.70071 (PMC12856796; doi:10.1002/jor.70071)
Supplement: Supplementary file 1 — Supplementary Tables 71125. [file JOR-44-na-s001.docx]

**Supplemental Table 1**: Baseline outcome measures between participants with imaging data or satisfaction data and those who do not. Presented as mean and standard deviation.

|  | Baseline imaging present | Baseline imaging not present | P value |
| --- | --- | --- | --- |
| Pain (NRS 0:10) | 2.8 | 3.6 | 0.215 |
| Function (FAAM-ADL) | 81.64 | 75.86 | 0.290 |
| Kinesiophobia (TSK-17) | 33.2 | 34.4 | 0.574 |
| Catastrophizing (PCS) | 5.2 | 6.8 | 0.525 |
|  | GROC/PASS  present | GROC/PASS not present | P value |
| Pain (NRS 0:10) | 2.8 | 3.7 | 0.215 |
| Function (FAAM-ADL) | 81.6 | 75.9 | 0.290 |
| Kinesiophobia (TSK-17) | 33.2 | 34.4 | 0.574 |
| Catastrophizing (PCS) | 5.2 | 6.8 | 0.525 |

Numeric rating scale (NRS), Foot and Ankle Ability Measure – Activity Daily living (FAAM-ADL), Tampa Scale of Kinesiophobia (TSK-17), Pain Catastrophizing Scale (PCS).

**Supplemental Table 2:** β - coefficient and p-value for each imaging variable as predictors for changes in worst pain and FAAM-ADL from univariate analysis.

| Imaging Finding | β - Coefficient | | 95% Confidence Intervals | | *p-*value |
| --- | --- | --- | --- | --- | --- |
| Worst Pain (NRS): |  |  | |  | |
| Tendon Thickness | -2.1 | -6.31 - 2.13 | | 0.325 | |
| Retro-Calcaneal Bursitis | 0.82 | -0.80 - 2.48 | | 0.309 | |
| Superficial Bursitis | 1.1 | -0.73 - 2.94 | | 0.231 | |
| Neo-vascularization | -1.7 | -5.94 - 2.55 | | 0.426 | |
| Intra-tendinous Calcification | 0.91 | -0.89 - 2.71 | | 0.318 | |
| Posterior-calcaneal spur | -0.7 | -2.66 - 1.23 | | 0.465 | |
| Haglund’s Deformity | 0.20 | -1.46 -1.87 | | 0.807 | |
| Widespread Pathology score | 0.18 | -0.32 - 0.42 | | 0.768 | |
| FAAM-ADL |  |  | |  | |
| Tendon Thickness | -22.3 | -54.6 - 10.1 | | 0.173 | |
| Retro-Calcaneal Bursitis | -11.5 | -24.4 - 1.45 | | 0.08 | |
| Superficial Bursitis | -5.7 | -20.7 - 9.2 | | 0.445 | |
| Neo-vascularization | -8.7 | -42.1 - 24.6 | | 0.599 | |
| Intra-tendinous Calcification | -14.0 | -27.8 - -0.23 | | 0.046 | |
| Posterior-calcaneal spur | 2.2 | -13.2 - 17.6 | | 0.776 | |
| Haglund’s Deformity | -12.3 | -25.3 - 0.62 | | 0.061 | |
| Widespread Pathology score | -1.5 | -4.31 - 1.38 | | 0.305 | |

Numeric rating scale (NRS), Foot and Ankle Ability Measure – Activity Daily living (FAAM-ADL)
